# In Vitro Refolding of Vault-like Protein Nanocapsules with a Novel Scaffolding Mechanism

**DOI:** 10.3390/ijms27010396

**Published:** 2025-12-30

**Authors:** Gabriela Breen, Martin Gonzales, Gracemarie Yeh, Tyler Delyon, Clare McNeill, Anika Lenci, Stephen Thong, Rodney Burton

**Affiliations:** Department of Chemistry and Physics, Ave Maria University, Ave Maria, FL 34142, USA

**Keywords:** biochemistry, nanoparticles, protein refolding, vaults, scaffold, FRET, dynamic light scattering, DLS, TEM, transmission electron microscopy

## Abstract

We attempted the in vitro scaffold-coordinated refolding of denatured major vault protein monomers into assembled vault-like nanoparticles. DNA or hyaluronic acid-binding tags were added to the MVP monomers, allowing MVP to align rotationally and translationally along these linear molecules. This was proposed to mimic the polyribosome assembly in vivo. Tagged MVP variants were expressed in *E. coli* and purified under denaturing conditions. Dynamic light scattering showed the formation of nanoparticles with a hydrodynamic radius of ~26 nm, consistent with the formation of vault-like nanoparticles. This was confirmed by transmission electron microscopy, FRET analysis, and cargo loading of CFP-INT fusion. CFP- and YFP-tagged MVP showed FRET only in the presence of MVP with a DNA-binding tag. This is the first successful instance of bioengineering of homogenous and heterogeneous vault-like nanoparticles, and at a potentially much larger scale than current protocols.

## 1. Introduction

The vault shell is composed of 78 copies of the major vault protein (MVP) and is the largest known eukaryotic protein nanocapsule, with approximately 10,000 vaults present in human cells [1,2]. Despite decades of research since its initial discovery in 1986 [3], the direct function of the vault protein remains poorly characterized [3,4], although it has been correlated with immune system activation and cancer defense [5,6]. The vault is also an attractive structure for bioengineering as a drug delivery vehicle [7,8] because it has a larger cargo capacity than current viral delivery vehicles, with far less danger of immune system inflammation since it is of human origin [9]. In addition, it easily dissociates in a low-pH environment. The difficulty in studying and bioengineering vaults is directly linked to the difficulty of purifying vaults in sufficient quantities. Current standard vault purification protocols are long, costly, laborious, and prohibitive at scale [10,11,12,13,14], involving extraction from insect cells or yeast [15] via ultracentrifugation [5]. In addition, when assembled in vivo, the polyribosome-based assembly prohibits the bioengineering of heterogeneous vaults [12]. The only current successful labeling of vault nanoparticles has been through chemical modification post-purification [16].

The primary model for the physiological mechanism of vault formation involves the simultaneous translation of multiple MVP monomers on a polyribosome-mRNA complex [12]. This allows vaults to assemble co-translationally while being properly aligned by the ribosomes, both translationally and rotationally, to allow for efficient vault complex assembly. This biochemical mechanism explains why attempts to refold denatured MVP monomers into self-assembled vault shells in vitro have been unsuccessful [11]. Without the polyribosome scaffold limiting the MVP degrees of freedom, vault self-assembly is entropically less favorable. Polyribosome-based assembly in vivo also requires that any protein sequence change in an MVP monomer must be present in all 78 MVPs of a given vault. This limits bioengineering, as N- and C-terminal protein tags easily become structurally disruptive when present in such large quantities [12].

We hypothesized that the polyribosome assembly mechanism could be mimicked in vitro by attaching fully translated MVP proteins to a linear scaffold using a scaffold-binding tag to form vault-like nanoparticles (VLPs). This was tested against two alternative linear scaffolds and three different scaffold-binding tags linked to MVP monomers. While not necessarily identical to canonical vaults, this approach could be used to bioengineer hollow VLPs of similar size and function.

Sso7d from the thermophilic *Saccharolobus solfataricus* was used as a C-terminal DNA-binding tag for MVP refolding on a DNA scaffold. Sso7d binds DNA with a small footprint of 4–5 bp [17], allowing for close alignment of the MVP monomers next to each other. Sso7d has also been shown to act as a refolding tag with the ability to revert protein aggregation [18].

Protamine was used as an N-terminal DNA-binding tag for MVP scaffold-based refolding. Protamine was chosen based on its theorized ability to unite vault halves through the simultaneous binding of two halves to the same DNA strand via the protamine (Prof. Leonard Rome, personal communication). Protamine is also observed to bend DNA into a loop [19], facilitating vault packaging. Protamine-tagged MVP is hereafter referred to as MARY-MVP.

However, N-terminal tags have also been shown to disrupt vault structure and may be less ideal as a scaffold [12].

To test for potential DNA-specific artifacts, we tested an alternate scaffold-tag pair: the hyaluronic acid (HA) scaffold and MVP with the HA-binding tag HABP35 [20,21]. HA is a linear carbohydrate polymer, and HABP35 is a small 29-residue peptide derived from the RHAMM HA-binding receptor. Analogous to the Sso7d, this is proposed to provide scaffold binding with a small footprint.

Two MVP tags, SUMO and GFP, with solubility-enhancing and/or entropic bristle [22,23] capability, were used to investigate if these could facilitate VLP formation in the absence of any scaffold binding. An untagged MVP was also used as a negative control.

Formation of heterogeneous VLPs was tested using FRET spectroscopy on a mixture of MARY-MVP, MVP-YFP, and MVP-CFP.

Refolding vault MVPs purified from *E. coli* to assemble VLPs in vitro is the first method for bioengineering of heterogeneous vault-like nanoparticles at a much larger scale than current protocols based on purification from insect cells.

## 2. Results

### 2.1. Purification of MVP and Tagged MVP Variants

**Figure 1:**
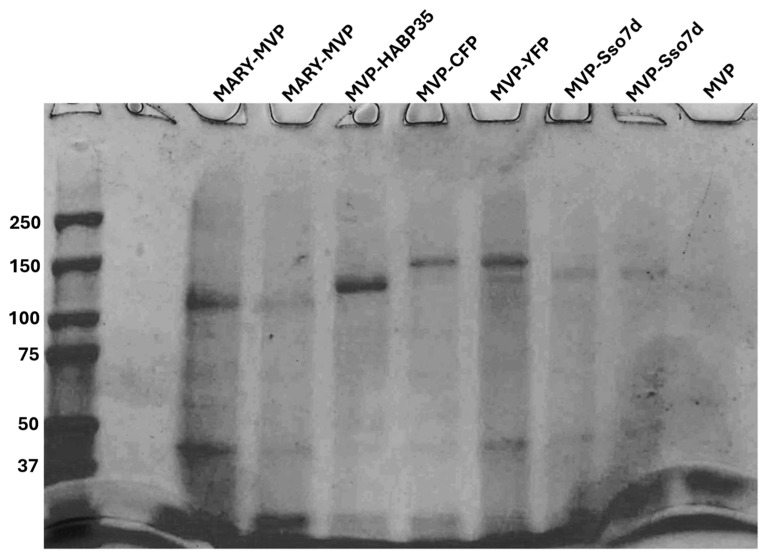
SDS-PAGE of purified MVP and tagged MVP variants. From left to right: MARY-MVP elution fraction 1, MARY-MVP elution fraction 2, MVP-HABP35 elution, MVP-CFP elution, MVP-YFP elution, MVP-Sso7d elution fraction 1, MVP-Sso7d elution fraction 2, and MVP with only the 6-His tag.

Human MVP and all tagged variants were grown in BL21-DE3 *E coli* and lysed with 6M Gdn-HCl. MVP and variants were purified using single-step Co-NTA resin under denaturing conditions with 8 M urea (See Figure 1). The final concentrations were determined by SDS-PAGE densitometry with ImageJ 1.54d using a BSA ladder. SDS-PAGE was also used to assess purity.

We tested two DNA scaffold-binding tags for the MVP: Sso7d on the C terminus (MVP-Sso7d) and protamine on the N-terminus (MARY-MVP). In addition, we generated an MVP with a C-terminal HA-binding tag, HABP35 (MVP-HABP35) [20,21]. All MVP variants were cloned into the pet28 plasmid (Kanr). Tag sequences were added to the MVP C or N-terminus with a linker consisting of a long poly-G sequence with a TEV protease cut site. All MVPs, including “untagged”, were also cloned with a 6-His affinity tag.

### 2.2. Dynamic Light Scattering Analysis

Time-resolved DLS was performed to observe the dynamic assembly process of VLPs. DLS measurements were performed in a 100 mM sodium phosphate buffer (pH 6.5–7.0) containing 50–100 mM NaCl, 1.5 mM MgCl_2_, and 27–400 mM urea. MVP was maintained at 1.8–8.0 nm. Initial refolding was consistently performed at 22 °C, followed by 4 °C for 1–2 days if applicable.

**Figure 2:**
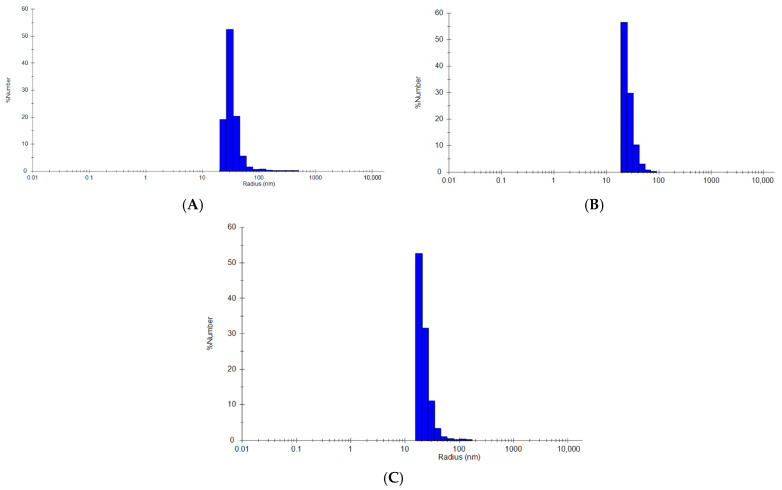
Dynamic light scattering representative histogram plots. (**A**) Representative completed refolding reaction of MVP-Sso7d; (**B**) representative completed refolding reaction of MARY-MVP; (**C**) representative completed refolding reaction of MVP-HABP35. All plots from Wyatt DYNAMICS 8 software.

**Figure 3:**
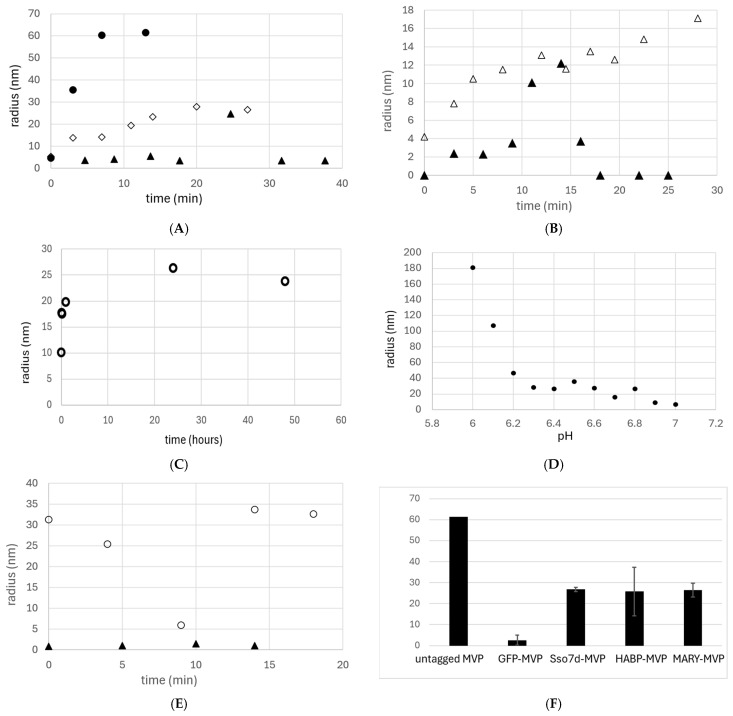
DLS results used to determine the hydrodynamic radius (nm) of MVP protein during refolding over time (min). (**A**) untagged MVP (●), MVP-GFP (▲), and Sso7d with 1.2 mg/mL copurified DNA (◇); (**B**) Sso7d-MVP vault formation with the addition of universal nuclease (+UN ▲) vs. control without universal nuclease (−UN △), both with 1.2 mg/ml copurified DNA; (**C**) time-resolved refolding of MARY-MVP in absence of nuclease (○); (**D**) refolding of MARY-MVP across a pH gradient; (**E**) HABP35-MVP vault formation: 8 nm HABP35-MVP in the presence of 1 nm HA (○) or absence of HA (▲); (**F**) summary of DLS radii of vesicles formed from negative controls, untagged MVP and GFP-MVP (grey bars), and from MVP with various scaffold-binding tags (black bars). The untagged MVP value was from n = 2 and lacks a standard deviation.

Refolding of Sso7d-tagged MVP in the presence of copurified DNA (1.2 mg/mL) resulted in the assembly of a complex with a 26.8 nm hydrodynamic radius within 20 min (Figure 2A and Figure 3A,B,F). This radius is consistent with previously reported DLS assays on intact vaults purified from eukaryotic cells (20–27 nm) [16,24]. To confirm the necessity of copurified DNA, Sso7d-MVP treated with a universal nuclease enzyme (UN+) to degrade any copurified DNA/RNA prior to refolding showed no significant nanocapsule formation (Figure 3B).

Refolding of MARY-MVP with copurified DNA over two days resulted in the formation of particles similar to those observed with the Sso7d tag (hydrodynamic radius = 26.4 nm) (Figure 2B and Figure 3C,D). Assembly of this VLP was observed to be highly pH-dependent, remaining monomeric above pH 6.8 and forming large aggregates below pH 6.2 (Figure 3D).

A 50 kDa HA scaffold at 1 nm was used with 8 nm MVP-HABP35 under the buffer conditions described above for Sso7d-MVP. Refolding into vault-sized nanocapsules (hydrodynamic radius = 30.75 nm) was observed only in the presence of exogenously added HA (Figure 2C and Figure 3E,F). In contrast to the other tagged variants, for the HABP35-MVP, nanocapsule formation was immediate, with no significant change over time observed through DLS (Figure 3E).

Wild-type MVP with no scaffold-binding tag showed rapid assembly of large bodies with a radius of ~60 nm (Figure 3A,F). The SUMO-MVP variant showed no DLS signal above the background, and GFP-MVP showed no significant nanoparticle formation (Figure 3A). DLS with only aggregates was recorded as zero. All linker regions between MVP and the C-terminal tags were identical, except for the GFP tag, which used a truncated linker.

DLS controls with the scaffold alone in refolding buffer were also run. To substitute for copurified DNA of unknown length, controls were run at 30 ng/μL, using either salmon genomic DNA or a pESC-leu2d purified plasmid of 11,042 bp. HA control was run using 1 nm 50 kD MW HA, as in refolding experiments. All controls using only scaffold showed no DLS peaks other than high-molecular-weight species of radii 254.5 nm (salmon DNA), 223.5 nm (plasmid DNA), and 255 nm (HA).

Results shown in Figure 2 are typical for all VLP samples, where only one major peak is observed with a polydispersity of ~25–30%. Due to the hollow interior cavity of the vault, reporting species percentages based on “percent mass” is unreliable, and “percent number” is used for all samples. The MIE spheres algorithm is also used in all samples.

### 2.3. Transmission Electron Microscopy Imaging

**Figure 4:**
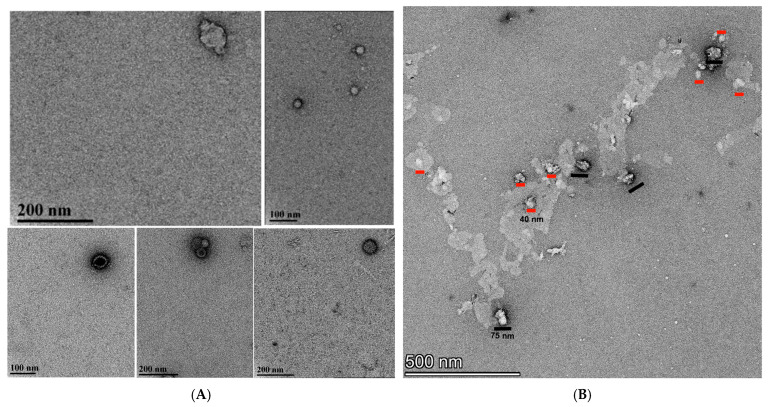

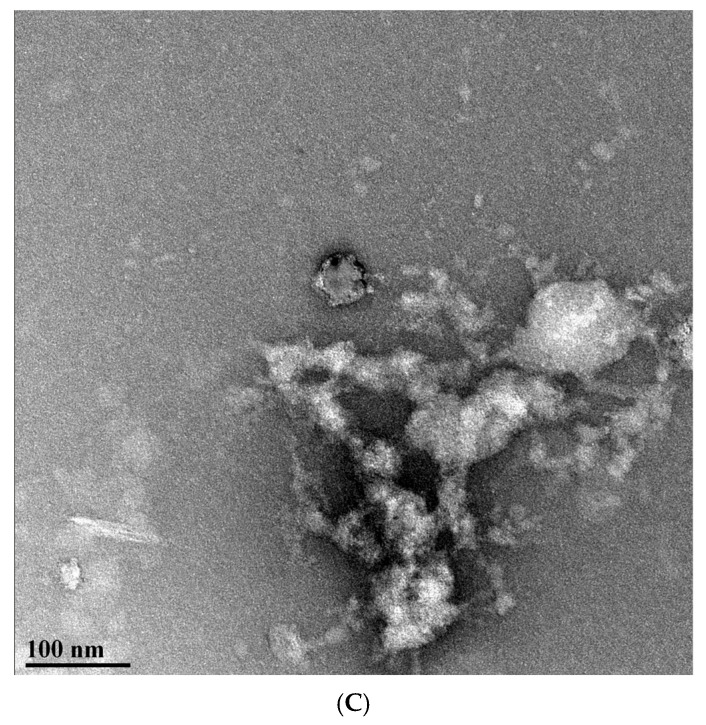
(**A**) TEM of VLPs formed with Sso7d-MVP scaffold-binding tag. (**B**) TEM of VLPs formed with MARY-MVP. Black scale bars are 75 nm, and red scale bars are 40 nm in length. (**C**) Large irregular vesicle-like disordered complexes formed with untagged MVP.

TEM images of refolded Sso7d-tagged, MARY-MVP-tagged, and untagged MVP were collected to determine the shape of VLPs observed in DLS (Figure 4). Samples were refolded as described above and concentrated. Sso7d-tagged MVP was fixed with 2% formaldehyde prior to concentration. A total of 10 µL of suspension was added to each grid for 5 min, followed by washing with ultrapure water. Samples were then stained with 1% uranyl acetate for 30 s before examination with a FEI Tecnai G2 Spirit Twin TEM.

A distribution of different sizes was observed for MVP-Sso7d. For MVP-Sso7d, the most common species observed was a circular nanocapsule, consistent with a top–down view of a “half vault” [12,24,25,26] (Figure 4A). Image analysis shows that 23% of these VLPs were ~27 nm in diameter, 38% were ~42 nm, and 38% were ~57 nm, consistent with DLS observations (Figure 4A), with rare full vaults being observed across multiple samples (top, left, n = 1). The nanocapsule diameters of (45 ± 13 nm) are consistent in size and shape with intact half-vaults previously purified from insect cells (41 nm) [12].

MARY-MVP was prepared similarly to MVP-Sso7d but in the absence of formaldehyde. The image of MARY-MVP refolded VLPs (Figure 4B) also shows a mixture of sizes consistent with full vaults (~40 nm by ~75 nm) and half vaults (~40 nm diameter); however, with a larger percentage of full VLPs (n = 4) vs. half (n = 8) compared to the experiment with MVP-Sso7d. However, the MARY-MVP VLPs appeared to be deformed relative to Sso7d, consistent with the understanding that the N-terminally tagged vault has decreased structural stability due to an increased tendency to unroll into sheets [12].

Control wild-type untagged MVP yielded large amorphous particle-like structures consistent with MVP renaturation without a scaffold, leading to damage and/or disordered aggregation [12] (Figure 4C). No separate VLP structures could be distinguished from the TEM.

### 2.4. Heterogeneous Vault Formation and Cargo Loading

**Figure 5:**
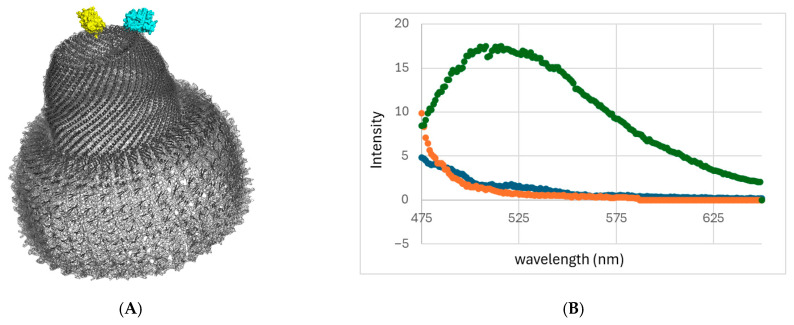

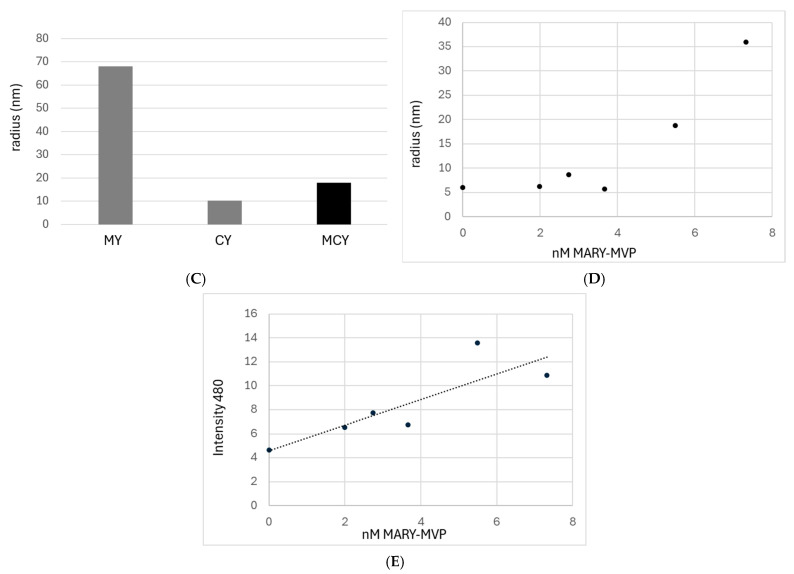
FRET and DLS analysis of heterogeneous VLPs. (**A**) Rendering of heterogeneous vault particles with CFP-MVP and YFP-MVP co-inserted with MARY-MVP. (**B**) Fluorescence intensity of FRET on VLPs produced by refolding denatured MVP variants. Solution MCY (green), control solution MY (orange), and control solution CY (blue). l_ex_ = 433 nm. (**C**) DLS hydrodynamic radii of solutions MCY, MY, and CY. (**D**) DLS hydrodynamic radii of varying concentrations of MARY-MVP at a fixed 8.3 nM concentration of CFP-INT. (**E**) Peak fluorescence emission intensity at 480 nm of CFP-INT of varying concentrations of MARY-MVP at a fixed 8.3 nM concentration of CFP-INT. l_ex_ = 433 nm. Dashed line is linear trendline fit.

FRET qualitative analysis was used to test the ability to form heterogeneous VLPs using the scaffold-based refolding protocol. MVP variants were produced with either yellow fluorescence protein (YFP) or cyan fluorescence protein (CFP) tags on their C-termini, MVP-YFP and MVP-CFP, respectively. Given the vault diameter of ~10 nm at the tip of the cap and total vault height of 75 nm, a CFP-YFP FRET signal should only be observable within vault halves and not between halves (Figure 5A).

MVP-CFP, MVP-YFP, and INT-CFP were purified under denaturing conditions, as described above, and the following three differing refolding solutions were prepared. Control solution MY: MARY-MVP (1.8 nM) was refolded with MVP-YFP (2.4 nm). Solution MCY: MARY-MVP (1.8 nm) was refolded with a combination of both MVP-YFP and MVP-CFP (1.2 nm each; Figure 5A. Control solution CY: MVP-YFP and MVP-CFP (1.2 nm each) were refolded in the absence of MARY-MVP. These three solutions were refolded in 20 mM phosphate, pH 6.6, 50 mM NaCl, and 1.5 mM Mg Cl_2_. The solutions were allowed to refold at room temperature for 20–30 min prior to concentration 57–150 fold through ultrafiltration (MWCO 100,000 kDa) and emission spectrum analysis. Upon excitation at the CFP excitation wavelength (433 nm), YFP fluorescence at 530 nm was detected only in solution MCY (Figure 5B), indicating a FRET-based signal from a heterogeneous complex. The spectrum of the buffer alone was subtracted from all signals. Consistent with expectations, no fluorescence was detected in either control solution (MY (no CFP) or CY (no scaffold-binding MVP)).

DLS was used to analyze the heterogeneous refolding solutions. Following concentration, solutions MCY, MY, and CY showed hydrodynamic radii of 18 nm, 68 nm, and 10.3 nm, respectively (Figure 5C). The 18 nm radius from solution MCY is more consistent with a half-vault than with a full vault. Half-vaults may be favored here due to the use of only 40% MARY-MVP necessary for optimal FRET signal–noise ratio. The 68 nm radius from solution MY may indicate a tendency of YFP to form oligomers at the locally high concentrations caused by the scaffolding. The 10.3 nm radius observed from solution CY, combined with the lack of any FRET signal, is consistent with MVP monomers that are unable to form nanoparticles.

The vault-sized particles, observed with DLS and TEM, along with MVP-YFP FRET with MVP-CFP, could be explained by a misfolded MVP aggregate. An INT-CFP fusion protein was therefore generated to test the cargo loading capability of VLP. If the VLPs are MVP aggregate, burying of the INT binding domain, or aggregation-caused quenching of the CFP, would result in a significant drop in fluorescence signal of INT-CFP bound to the VLP. The INT domain is a well-established fusion tag to load cargo proteins to the interior hollow cavity of the vault complex [9]. The standard INT-binding site faces the vault interior, spans three domains of the MVP [27], and is ~30 nm from the vault cap.

INT-CFP binding to refolded VLPs was examined with an ultrafiltration fluorescence binding assay, as in Kickhoefer et al. (2005) [8]. Refolding was conducted as above for MARY-MVP in pH 6.5 phosphate buffer, 50 mM NaCl, 1.5 mM MgCl_2_. INT-CFP was simultaneously refolded with MARY-MVP and was held fixed at 8.28 nm as [MARY-MVP] was increased from 0 to 7.3 nM. After refolding for 20 min, refolding solutions were centrifugally concentrated (MWCO 100 kDa; INT-CFP MW 45.2 kDa) from 15 mL to ~150 μL. Concentrated refolding reactions were monitored for CFP fluorescence by excitation at 433 nm, and emission peak was monitored at 480 nm (Figure 5E). DLS radii were also collected as above for each MARY-MVP concentration (Figure 5D).

As shown in Figure 5E, a linear increase in CFP fluorescence is observed as the [MARY-MVP] is increased. At ~5 nm [MARY-MVP], DLS observes a sharp transition from monomeric MARY-MVP to VLP formation (Figure 5D). This inflection point in radius is not associated with a concordant deviation in CFP emission intensity, but instead, CFP fluorescence continues to increase linearly as a function of [MARY-MVP]. This result is consistent with previous studies loading GFP into the hollow vault interior, where fluorescence is preserved upon loading [8].

## 3. Conclusions

The proposed scaffold-based refolding mechanism is consistent with our results. The precise conformation of the VLPs, whether nanoparticles of the canonical vault shape or a variation of the vault, will be a matter of future study. However, MVP-Sso7d, MARY-MVP, and MVP-HABP35 with scaffold-binding tags all showed DLS signal consistent with formation of vault-sized nanoparticles. The MVP-Sso7d VLP formation was eliminated with the addition of nuclease, consistent with reliance on a DNA scaffold. HABP35-MVP VLPs also formed only in the presence of the HA scaffold. Neither SUMO-MVP nor GFP-MVP formed nanoparticles under the same conditions because of their inability to bind the DNA scaffold. Using TEM with MVP-Sso7d, a significant number of half-vault-like particles were observed, with one observed full vault. MARY-MVP, meanwhile, showed a significant number of VLPs consistent in size with both half and full-vaults. However, these were somewhat broken compared to MVP-Sso7d structures. In a future study, it will be worthwhile to have a mixture of MVP-Sso7d and MARY-MVP. This may provide both well-ordered VLPs from the Sso7d tag while allowing MARY-MVP to link them together to allow a higher percentage of full vaults.

Formation of VLPs using heterogeneously tagged MVPs was tested using FRET. A FRET signal was observed between the MVP-tagged donor-acceptor pair in the presence of scaffold-binding MARY-MVP. This is consistent with proper alignment of the C-termini in the heterogeneous VLPs. CFP fluorescence intensity of INT-CFP fusion protein showed no significant deviation in intensity upon binding free MVP vs. binding MVP as part of a 25 nm radius VLP. This is consistent with proper refolding of the three-domain INT binding site. This is also consistent with no loss of CFP signal due to aggregation-caused quenching or burying of the INT binding site as the VLP forms. The data are consistent with intact INT-binding sites on the VLPs with a hollow cavity [8,27].

This is the first instance of VLP formation in vitro using refolded MVP monomers, and to the authors’ knowledge, this is the first use of a linear scaffold for the guided refolding of any multimeric complex. The in vitro assembly eliminates the need for the high-cost, low-yield production from eukaryotes, and the single-step affinity purification from *E. coli* allows for low-cost, large-scale purification. This protocol may be expanded to bioengineering applications such as heterogeneous VLPs with both cell-localization and therapeutic tags for drug delivery.

## 4. Materials and Methods

### 4.1. Transmission Electron Microscopy

TEM was used to visualize the refolded vault protein nanocapsules. The refolded samples were fixed with 2% formaldehyde in refolding buffer at room temperature prior to shipping to University of Florida for analysis. Glow-discharged carbon-coated 400 mesh copper grids (CF400CU, Electron Microscopy Sciences, Hatfield, PA, USA) were floated onto 10 µL of suspension for 5 min. The excess solution was blotted from the grid with filter paper. The sample was washed by touching the grid to drops of ultrapure water (3×) and floated onto a drop of 1% aqueous uranyl acetate for 30 s, blotted dry, and examined with a FEI Tecnai G2 Spirit Twin TEM (FEI Corp., Hillsboro, OR, USA), and digital images were acquired with a Gatan UltraScan 2k × 2k camera and Digital Micrograph software (Gatan Inc., Pleasanton, CA, USA). Some samples were visualized with a ThermoFisher G2 Talos L120C TEM (Thermo Fisher Corp., Wilham, MA, USA) operated at 120 kV, and digital images were acquired with a Ceta CMOS 4K × 4K camera and Velox software ThermoFisher G2 Talos L120C TEM (Thermo Fisher Corp., Wilham, MA, USA).

### 4.2. Transformation and Growth of E. coli

Genes for variants of tagged MVP variant plasmids were made by GenScript. All were cloned into Kanr Pet28. These plasmids were transformed into competent BL21-DE3 E. coli cells according to the New England Biolabs protocol [28] and grown on LB agar plates in a 37 °C incubator for ~18 h. Colonies were then introduced into seed culture solutions of 50 mL of LB Broth and 50 µL of 50 mg/mL kanamycin (1:1000 dilution of Kanamycin) [28]. The cultures were placed in a shaking incubator for approximately 16 h at 37 °C. The seed cultures were then poured into larger cultures of 1 L LB broth and 1 mL of 50 mg/mL kanamycin and allowed to grow at 37 °C in a shaking incubator. The growth of E. coli was periodically checked using a PerkinElmer UV/VIS until the optical density (OD) readings at 600 nm were within 0.5–0.8. Then, 1 mL of 0.8M IPTG was added to each 1 L culture to stop bacterial growth and to induce production of the desired protein [29]. The cell cultures were placed overnight in a shaking incubator at 25 °C and harvested by centrifugation at 5000 rpm for 10 min. The supernatant was discarded, and the pellet was harvested and stored at −80 °C until purification.

### 4.3. Protein Purification

Recombinant human MVP proteins and INT-CFP fusion were purified under denaturing conditions using the Ni-NTA Purification System protocol from Thermo Fisher Scientific [29]. The frozen cell pellet collected from centrifugation was thawed either on ice or in the fridge and then solubilized at 37 °C, pH 7.8, in Guanidinium Lysis Buffer [29] to produce a final 40 mL of lysate. The lysate was then sonicated using a Branson Sonifier Cell Disruptor 200 at a high-intensity setting with 50% on/off bursts for 12 min.

Prior to the purification process, the columns were charged by running 15 mL of DI water, 10 mL of 0.05M EDTA, 10 mL of DI water, 10 mL of 0.05M cobalt, another 10 mL of DI water, and finally 10 mL of pH 7.8 8M urea binding buffer [29] through the column resin. The batch purification method was used to bind the protein to the column beads as follows: the cobalt beads in the column were resuspended in the binding buffer within the column. The beads and residual binding buffer from the column were then allocated into two 15 mL FALCON tubes along with the prepared lysate before being centrifuged at ~1000 rpm for 30 s. The supernatant was aspirated before resuspending the beads in additional binding buffer. The beads were gently centrifuged under the same conditions, and the supernatant was aspirated. The beads were then resuspended in residual binding buffer and added back to the column for the continuation of the Ni-NTA Purification protocol [29].

Stocks A and B (sodium phosphate monobasic and dibasic, respectively) were prepared without 5M NaCl. A separate 5M NaCl solution was prepared, and the appropriate amount was added directly to the buffers needed for denatured purification. Outside this exception, buffers were prepared according to the protocol instructions. A 5.3 pH wash buffer was produced or used during the purification process. A total of ~5 mL of pH 7.8 binding buffer was run through the column and collected in a waste beaker. Wash buffer (6 mL, pH 6.0) was added to the column and collected in 1 mL fractions in microfuge tubes. Finally, 8 mL of elution buffer (pH 4.0) was added to the column and collected in 1 mL fractions in microfuge tubes. Following collection of the eighth fraction, an additional elution buffer was added to the column to prevent the beads from drying. The column and collected fractions were stored at 4 °C. For all MVP variants, concentration was determined using densitometry through the software ImageJ 1.54d by comparing a scale of varying BSA concentrations against MVP bands. Sso7d-MVP was determined to contain 1.2 mg/mL copurified DNA by Quanti-IT Picogreen assay from Invitrogen.

Sso7d tag sequence: TVKFKYKGEEKEVDISKIKKVWRVGKMISFTYDEGGGKTGRGAVSEKD

APKELLQMLEKQKKGGGGGGSLVPRGSGGGGGHHHHHH.

Linker sequence: GGGGGSLVPRGSGGGGSENLYFQSGGGGSLVPRGSGGGGGGGGGGGGGGG.

HABP35 tag sequence: LKQKIKHVVKLKVVVKLRSQLVKRKQNGGGGGGSLVPRGSGGGGGHHHHHH.

MARY MVP tag and linker sequence: MARYRCCRSQSRSRYYRQRQRSRRRRRRS CQTRRRAMRCCRPRYRPRCRRHGGGGGG.

Superfolder Cyan fluorescent protein tag sequence: GGGGSGGGGSGGGGSMSKGEE

LFTGVVPILVELDGDVNGHKFSVRGEGEGDATNGKLTLKFICTTGKLPVPWPTLVTTLTWGVQCFSRYPDHMKRHDFFKSAMPEGYVQERTISFKDDGTYKTRAEVKFEGDTLVNRIELKGIDFKEDGNILGHKLEYNFNSHNVYITADKQKNGIKANFKIRHNVEDGSVQLADHYQQNTPIGDGPVLLPDNHYLSTQSVLSKDPNEKRDHMVLLEFVTAAGITHGMDELYKGGGGGGHHHHHH.

Superfolder Yellow fluorescent tag protein sequence: GGGGGGGGGGGGGGGMSKGEELF TGVVPILVELDGDVNGHKFSVRGEGEGDATNGKLTLKFICTTGKLPVPWPTLVTTLTYGVQCFSRYPDHMKRHDFFKSAMPEGYVQERTISFKDDGTYKTRAEVKFEGDTLVNRIELKGIDFKEDGNILGHKLEYNFNSHNVYITADKQKNGIKANFKIRHNVEDGSVQLADHYQQNTPIGDGPVLLPDNHYLSYQSVLSKDPNEKRDHMVLLEFVTAAGITHGMDELYKGGGGGGHHHHHH.

### 4.4. Refolding and Dynamic Light Scattering Experiments

Experimentation of the refolding of the vault protein using a linear scaffold was performed by mixing varying amounts of refolding buffer (100 mM sodium phosphate, pH 7.0, containing 100 mM NaCl and 1.5 mM MgCl_2_), DNA or HA at varying concentrations, and purified denatured MVP in the cuvette of the Wyatt dynamic light scattering (DLS) nanostar instrument. The sample was then monitored in the DLS. Changes in the species within the solution over time were also monitored using the DYNAMICS software.

The mixing of the refolding process was as follows: 320 μL of refolding buffer was added to the DLS cuvette, followed by 1.1–16 μL of tagged MVP. Sso7d-MVP or MARY-MVP for DNA scaffold and HABP35-MVP for HA scaffold were added to the cuvette at 1.8–8 nM and mixed by pipetting. The final volume added to the cuvette was 40 μL.

All DLS assays were conducted at 25 °C, with a 5 s acquisition time and averages of 25–50 acquisitions. The model for radius calculation was MIE spheres, with %Number readout. The correlation cutoff was 1.5 to 2 ×10^5^ μs, low radius cutoff was 3.0 nm, and high radius cutoff was 10,000 nm.

### 4.5. Nuclease Controls: Eliminating Potential Contaminating DNA

Sso7d (8M urea) was incubated with or without universal nuclease (UN+ and UN−, respectively) stock (250 U/mL nuclease) at a 3:1 buffer volume ratio for 10 min to remove any copurified DNA/RNA polymers. UN+ samples were incubated with nuclease buffer containing 50% glycerol.

### 4.6. Heterogeneous Vault Formation

MARY-MVP (1.8 nm) was refolded with MVP-YFP (2.4 nm) or a combination of both MVP-YFP and MVP-CFP (1.2 nm each). In addition, MVP-YFP and MVP-CFP (1.2 nm each) were refolded in the absence of MARY-MVP. These three solutions were refolded in 20 mM phosphate, pH 6.6, 50 mM NaCl, 1.5 mM Mg Cl_2_. Solutions were allowed to refold at room temperature for 20–30 min prior to concentration 57–150 fold through ultrafiltration (MWCO 100,000 kDa). FRET data were collected on a Varian Cary Eclipse fluorometer with an excitation wavelength of 433 nm and 20 nm excitation and emission slits.

### 4.7. INT-CFP Cargo Loading

INT-CFP binding to VLPs was examined with an ultrafiltration fluorescence binding assay, as in Kickhoefer et al. (2005) [8]. MARY-MVP was refolded in pH 6.5 phosphate buffer, 50 mM NaCl, 1.5 mM MgCl_2_. 8.28 nM INT-CFP was held fixed, simultaneously refolded in the same solution as [MARY-MVP], and was increased from 0 to 7.3 nm. After refolding for 20 min, refolding solution was centrifugally concentrated (MWCO 100 kDa; INT-CFP) from 15 mL to ~150 μL. Concentrated refolding reactions were monitored for CFP fluorescence by excitation at 433 nm, and emission peak was monitored at 480 nm.

## Data Availability

The datasets used and/or analyzed during the current study are available from the corresponding author on reasonable request.
